# Long-Term Outcomes and Therapeutic Strategies for Newly Diagnosed Crohn’s Disease in the Biologic Era: A Nationwide Claims-Based Study from Japan

**DOI:** 10.31662/jmaj.2025-0438

**Published:** 2025-11-21

**Authors:** Rintaro Moroi, Yoichi Kakuta, Hideya Iwaki, Daisuke Okamoto, Hiroshi Nagai, Yusuke Shimoyama, Takeo Naito, Hisashi Shiga, Atsushi Masamune

**Affiliations:** 1 Division of Gastroenterology, Tohoku University Hospital, Sendai, Japan

**Keywords:** Crohn’s disease, biologics, steroids, pediatric patients, older patients

## Abstract

**Introduction:**

Biological agents have been approved and are now widely used in the treatment of Crohn’s disease (CD), fundamentally changing therapeutic strategies. However, evidence regarding long-term outcomes and treatment approaches―particularly in elderly onset (ED) and pediatric onset (PO) CD―remains limited. This study aimed to clarify the long-term prognosis and therapeutic patterns of patients newly diagnosed with CD using a nationwide real-world claims database in Japan.

**Methods:**

A retrospective cohort study was conducted using the DeSC Healthcare Inc. database, identifying 1,345 patients newly diagnosed with CD from 2014 to 2023. Patients were categorized by age at onset as pediatric (<16 years), elderly (≥65 years), and non-elderly/non-pediatric. Treatment strategies were classified as step-up or top-down based on the initial therapy. Kaplan-Meier analysis and log-rank tests were used to evaluate the cumulative rates of advanced therapy-free survival, steroid-free survival, and biological retention.

**Results:**

Of the 1,345 patients, 56 had PO and 472 had EO disease. Top-down therapy was used in 52.2% of cases. The 5-year advanced therapy-free and steroid-free rates were 57.2% and 44.2%, respectively. EO patients showed the highest advanced therapy-free rate (76.2%) and the lowest use of top-down therapy. PO patients had the lowest steroid-free rate and the highest top-down therapy (80%).

**Conclusions:**

Age at CD onset influences treatment strategies in Japan. Early biologic therapy is common in younger patients, whereas conservative approaches are preferred in older adults. Real-world data provide important insights for optimizing individualized CD management in the biological era.

## Introduction

Crohn’s disease (CD) is a chronic inflammatory bowel disease that affects the entire gastrointestinal tract and is characterized by recurrent exacerbations and remissions ^(1), (2)^. Advanced therapies, including biologics and small-molecule agents, have become available worldwide and have demonstrated high clinical efficacy ^(3)^. These agents have shifted therapeutic strategies and goals from clinical to endoscopic remission ^(4)^. In Japan, infliximab was first approved for the treatment of CD in 2002 (episodic administration), followed by adalimumab in 2010 and other drugs. We are currently in the biological era. Although step-up or top-down therapy is employed in clinical practice for CD, conflicting results regarding the optimal treatment have been reported ^(5), (6)^. Furthermore, evidence on long-term prognosis among patients treated with biologics―especially differences by age at CD onset―remains scarce. CD usually develops between the ages of 18 and 35 years ^(7)^, and pediatric onset (PO) or elderly onset (EO) disease is relatively rare. Clarifying these uncertainties is essential for guiding current clinical practice for patients with CD.

The number of newly developed CD cases in a single center, particularly PO or EO cases, was small. To address this issue, we conducted clinical research using a database generated by DeSC Healthcare, Inc. ^(8), (9), (10)^. The database comprises medical claim data and contains large-scale data, enabling analysis of many cases of CD across Japan. Therefore, the DeSC database is useful for analyzing rare scenarios, including PO and EO CD.

This study aimed to clarify differences in long-term prognosis and therapeutic strategies for new-onset CD cases in the biologic era.

## Materials and Methods

### Ethics approval and patient consent statement

The study protocol was reviewed and approved by the Ethics Committee of the Tohoku University Graduate School of Medicine (2022-1-412). The requirement for informed consent was waived because the patient data were anonymized.

### Study design and data source

This retrospective study used a large-scale claims database. Specifically, we analyzed a dataset comprising prescription claim receipts from patients with CD developed and maintained by DeSC Healthcare, Inc. Permission was obtained from DeSC Healthcare, Inc. to use their dataset for this research. The dataset contains anonymized inpatient and outpatient prescription claim records―including age, sex, medical treatment, and clinical diagnosis based on the International Classification of Diseases, 10th Revision (ICD-10), from a health insurance association in Japan from April 2014 to July 2023.

### Extraction of eligible patients and data collection

**Figure 1.**
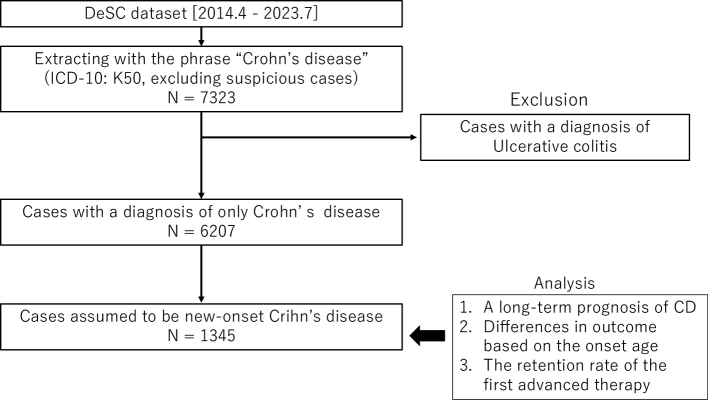
Flowchart for the extraction of eligible patients from the patient claims database of DeSC Healthcare, Inc. In total, 6,207 cases of new-onset CD were extracted. The long-term prognosis and differences in outcomes based on the age at onset and retention rate of the first advanced therapy were analyzed. CD: Crohn’s disease; ICD-10: International Classification of Diseases, 10th Revision.

Eligible patients were extracted from the DeSC Healthcare database (Figure 1). CD cases were identified using ICD-10 code K50, and entries flagged as suspicious (i.e., containing the word “suspicious”) were excluded. Patients with a clinical diagnosis of ulcerative colitis were also excluded. Subsequently, cases assumed to be newly developed CD were extracted based on the definitions described below.

We collected the following patient data from the DeSC dataset: sex, birth year and month, and observation start and end dates. Information on prescribed medications was also obtained, including 5-aminosalicylic acid (5-ASA); systemic steroid administration (prednisolone); immunomodulators (azathioprine and 6-mercaptopurine); and advanced therapies, including biologics (infliximab, adalimumab, ustekinumab, vedolizumab, and risankizumab) and small-molecule agents (upadacitinib).

### Definitions

A “new” prescription for each drug was defined as the absence of any prescription in the 26 weeks before the first prescription date of the medicine during the observation period. Other prescriptions were defined as “old” prescriptions.

Discontinuation was defined as the absence of a prescription for more than 13 weeks after the next scheduled prescription date (e.g., >21 weeks after the last infliximab infusion or >25 weeks after the last ustekinumab injection). The next scheduled prescription date differed by drug.

We defined new-onset CD as cases that had (1) a new prescription for 5-ASA, a systemic steroid, or an advanced therapy, and (2) no old prescriptions for these drugs during the observation period. Based on this assumption, the age of CD onset was estimated. Accordingly, patients who underwent surgery as the initial treatment before receiving any medical therapy could not be identified as new-onset CD in this study. The treatment approach was classified according to the initial prescription record. Patients who started with corticosteroids and/or 5-ASA were categorized as step-up, regardless of whether they subsequently received advanced therapies. Patients who started with advanced therapies (with or without corticosteroids) were categorized as top-down. If corticosteroids and advanced therapies were initiated simultaneously, the case was classified as top-down. Immunomodulators (azathioprine, 6-mercaptopurine, methotrexate) were not considered in this classification.

### Data analysis and statistics

The new CD development cases were divided into three categories depending on the estimated age of onset: EO, PO, and non-elderly, non-pediatric onset (NENP). PO was defined as age <16 years, based on the Montreal classification ^(11)^. The definition of EO was age ≥65 years, based on the World Health Organization classification ^(12)^.

This study evaluated the cumulative advanced therapy-free rate using the Kaplan-Meier method for long-term prognosis after new-onset CD. The impact of age at onset on long-term prognosis was also investigated using the log-rank test. A comparison of the initial advanced therapy retention rates was conducted.

This study subsequently evaluated the cumulative first advanced therapy retention and steroid-free rates and compared them among the age categories of CD onset.

The ratio of top-down therapy to step-up therapy was calculated for all patients and for each age category. Furthermore, a Cochrane-Armitage trend test was conducted to evaluate the correlation between the rate of top-down therapy and age categories.

The threshold for statistical significance was set at p < 0.05. All analyses were performed using the JMP Student Edition 18 (SAS Institute, Tokyo, Japan).

## Results

### Background of the study population

This study identified 1,345 cases that were assumed to have newly developed CD, based on the definitions described above (Figure 1). The characteristics of these cases are summarized in Table 1. The administration rates of systemic steroids and advanced therapy were 50.1% and 34.3%, respectively. Infliximab was the most frequently used advanced therapy, followed by adalimumab and ustekinumab. The mortality rate during the observation period was 7.2%. Table 2 shows detailed comparisons of follow-up duration across the onset-age categories. The median observation period was 1,856 days (interquartile range [IQR], 1,384-2,801). By onset age, it was 1,795 days (IQR, 1,430-2,435) for EO, 2,099 days (IQR, 1,278-2,891) for non-elderly/non-pediatric, and 2,419 days (IQR, 1,599-3,013) for pediatric patients, with significant differences among groups (p < 0.0001).

**Table 1.** Background of Newly Developed Patients with CD.

Variable	Newly developed patients with CD N = 1,345
Sex (male/female)	882/463
Age categories of CD-onset	
Elderly onset	472
Non-elderly, non-pediatric onset	817
Pediatric onset	56
Average observation period (SD)	1,949.2 (824.9) days
Use of systemic steroid	674 (50.1%)
Use of advanced therapy	462 (34.3%)
Breakdown of the initial advanced therapy	
Infliximab	183 (39.6%)
Adalimumab	158 (34.2%)
Ustekinumab	87 (18.8%)
Vedolizumab	32 (6.9%)
Upadacitinib	1 (0.21%)
Risankizumab	1 (0.21%)
Death	97 (7.2%)

CD: Crohn’s disease; SD: standard deviation.

**Table 2.** Comparisons of Median Observation Period Across Crohn’s Disease Onset-Age Categories.

Onset-age category	Overall N = 867	Elderly onset N = 272	Non-elderly, non-pediatric onset N = 550	Pediatric onset N = 45	p-Value
Median observation period (interquartile range), days	1,856 (1,384-2,801)	1,795 (1,430-2,435)	2,099 (1,278-2,891)	2,419 (1,599-3,013)	<0.0001*

*Median test.

### Long-term prognosis for new-onset CD

**Figure 2.**
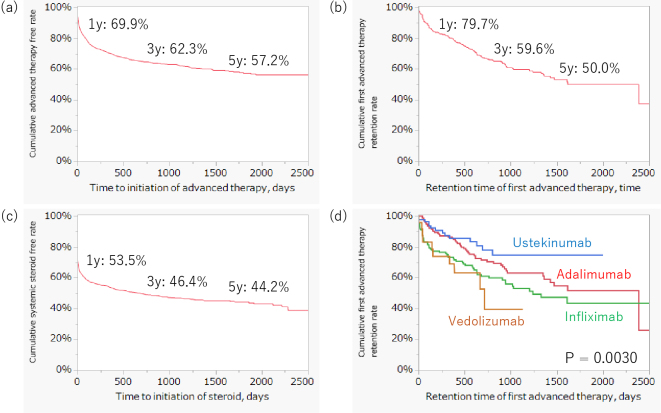
Kaplan-Meier curve describing the long-term prognosis of new-onset CD cases. (A) The cumulative advanced therapy-free rate in patients with new-onset CD was 57.2 % at 5 years post-onset of CD. (B) The cumulative first advanced therapy retention rate at 5 years was 50.0%. (C) The cumulative systemic steroid-free rate at 5 years was 44.2%. CD: Crohn’s disease.

The cumulative advanced therapy-free and systemic steroid-free rates at five years in patients with new-onset CD were 57.2 % and 44.2%, respectively (Figure 2A and C). The cumulative first advanced therapy retention rate at five years after CD onset was 50.0 % (Figure 2B). A comparison of the retention rates of the first advanced therapy with infliximab, adalimumab, ustekinumab, and vedolizumab is shown in Figure 2D. Ustekinumab demonstrated the best retention rate compared with the other advanced therapies (p = 0.0030).

**Figure 3.**
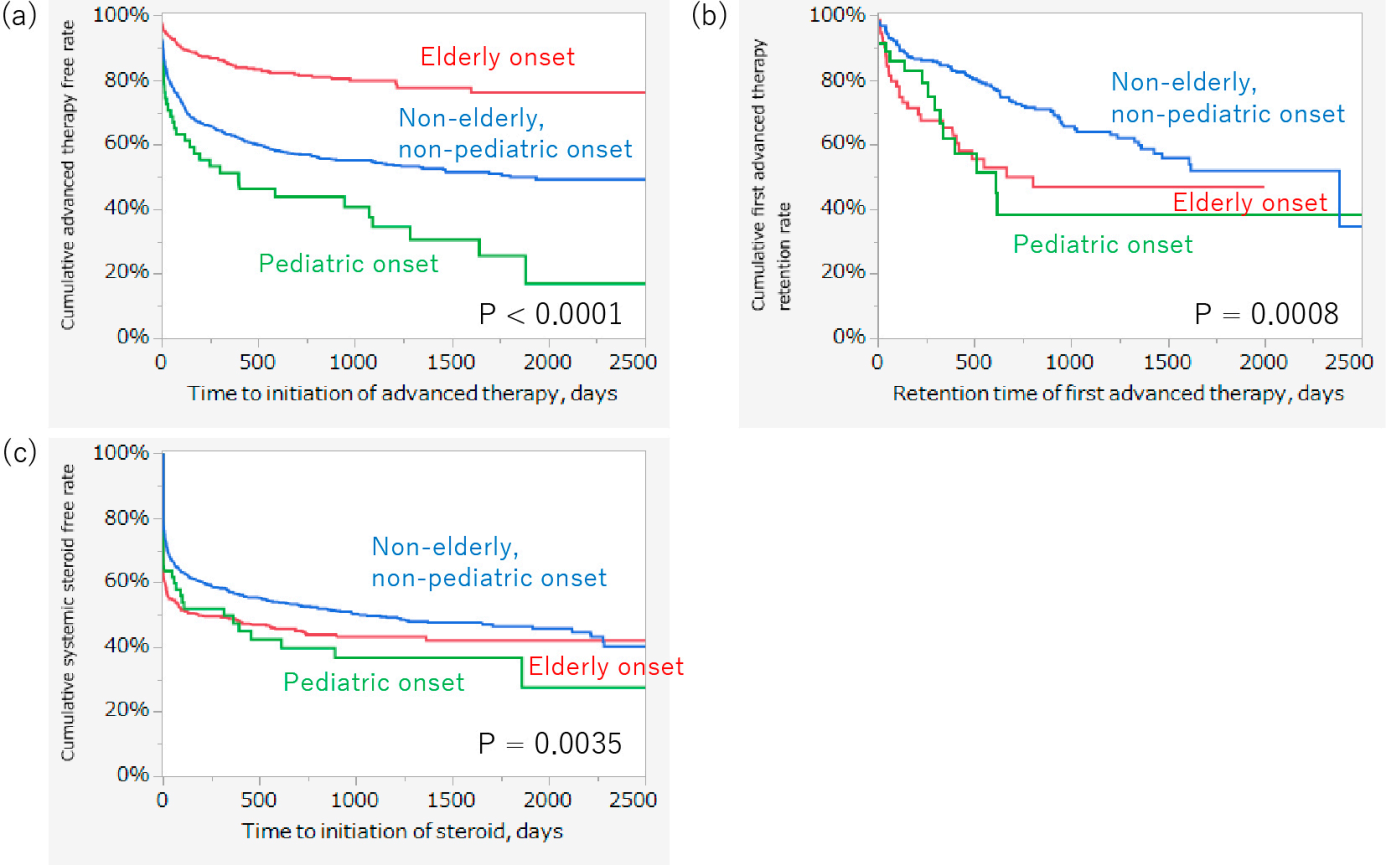
The Kaplan-Meier curve describing the differences in long-term prognosis between onset age categories. (A) The cumulative advanced therapy free rate of pediatric onset, non-elderly/non-pediatric onset, and elderly onset at 5 years was 17.0%, 49.9%, and 76.2%, respectively (p < 0.0001). (B) The cumulative first advanced therapy retention rate of pediatric onset, non-elderly/non-pediatric onset, and elderly onset at 5 years was 41.8 %, 51.9 %, and 46.8 %, respectively (p = 0.0008). (C) The cumulative systemic steroid-free rate of pediatric onset, non-elderly/non-pediatric onset, and elderly onset at 5 years was 36.7 %, 46.5 %, and 42.0 %, respectively (p = 0.0035).

Figure 3 shows the differences in the long-term prognosis between the onset age categories. The cumulative advanced therapy-free rate of EO CD patients at five years after CD onset (76.2%) was significantly higher than that of PO patients (17.0%) and NENP patients (49.9%) (p < 0.0001) (Figure 3A). The cumulative advanced therapy retention rate of NENP patients was also significantly higher than that of EO and PO patients with CD (p = 0.0008) (Figure 3B). The systemic steroid-free rate in PO CD patients was statistically lower than that in EO and NENP patients with CD (p = 0.0035) (Figure 3C).

### Comparison of the top-down and step-up therapy ratios among the CD onset age categories

**Figure 4.**
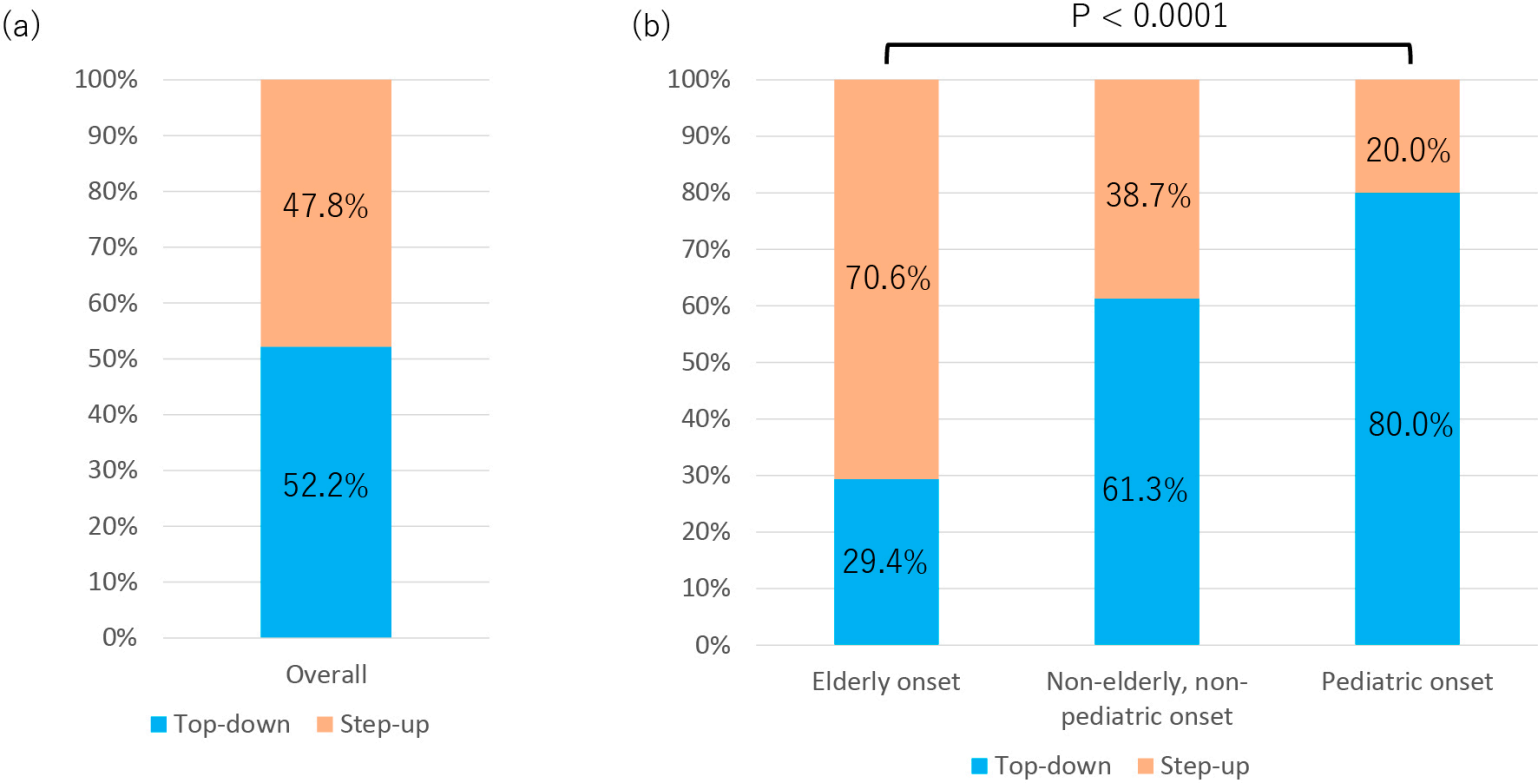
Comparison of top-down and step-up therapy. (A) Among all patients with Crohn’s disease, 52.2% received top-down therapy, while 47.8% received step-up therapy. (B) Comparison by age at disease onset. The proportion of patients treated with top-down therapy increased with younger onset age, whereas the proportion of patients treated with step-up therapy increased with older onset age (p < 0.0001).

Figure 4 and Table 3 show the rates of top-down and step-up therapies in all patients and each CD onset age category. The top-down rate in all patients was 52.2%, with a step-up rate of 47.8% (Figure 4A). As the age at onset increased, the rate of top-down therapy decreased (p < 0.0001)(Figure 4B). Infliximab and adalimumab were administered to the PO and NENP groups, whereas ustekinumab and vedolizumab tended to be selected for the EO group (p < 0.0001) (Table 3).

**Table 3.** Ratio of Top-Down Versus Step-Up Approaches and Breakdown of Initial Advanced Therapy Across Different Age of Onset Categories.

Onset-age category	Overall N = 867	Elderly onset N = 272	Non-elderly, non-pediatric onset N = 550	Pediatric onset N = 45	p-Value
Top-down: Step-up, n (%)	453 (52.2%): 414 (47.8%)	80 (29.4%): 192 (70.6%)	337 (61.3%): 213 (38.7%)	36 (80.0%): 9 (20.0%)	<0.0001
First advanced therapy					<0.0001
Infliximab	183 (39.6%)	25 (30.5%)	136 (39.5%)	22 (61.1%)	
Adalimumab	158 (34.2%)	17 (20.7%)	130 (37.8%)	11 (30.6%)	
Ustekinumab	87 (18.8%)	19 (23.2%)	65 (18.9%)	3 (8.3%)	
Vedolizumab	32 (6.9%)	20 (24.4%)	12 (3.5%)	0 (0%)	
Upadacitinib	1 (0.21%)	1 (1.2%)	0 (0%)	0 (0%)	
Risankizumab	1 (0.21%)	0 (0%)	1 (0.29%)	0 (0%)	

**Figure 5.**
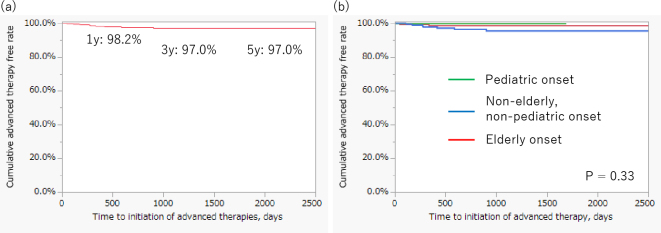
Long-term outcomes of patients treated with step-up therapy. (A) Cumulative advanced therapy-free rates at 1, 3, and 5 years after diagnosis in patients treated with a step-up strategy were 98.2%, 97.0%, and 97.0%, respectively. (B) No significant differences in advanced therapy-free survival were observed among the age-at-onset groups.

Figure 5A shows the long-term outcomes of patients who underwent the step-up strategy. The cumulative advanced therapy-free rates at one, three, and five years were 98.2%, 97.0%, and 97.0%, respectively. There was no difference in the cumulative advanced therapy-free rate among the onset age categories in patients treated with the step-up strategy (Figure 5B).

## Discussion

The long-term outcomes after new-onset CD were estimated using a dataset based on a nationwide medical claims database. This study analyzed 1,345 cases of new-onset CD, including 472 EO and 56 PO cases, although these were presumptively identified cases. This is one of the study’s advantages. The dataset used in this study contains both inpatient and outpatient data from across Japan, regardless of the referral center. Therefore, these results are expected to reflect real-world healthcare scenarios in Japan regarding patients with CD. This is an important aspect of the present study.

Results indicate that physicians in Japan should consider a patient’s age and condition when selecting appropriate treatment strategies and medications. In short, the “Treat to Target” strategy ^(4), (13)^ based on patients’ age at onset is well conducted in the clinical practice of Inflammatory Bowel Disease in Japan. In the all-age categorical analysis, the top-down rate was 52.2%. Furthermore, over 40% of patients with CD had undergone advanced therapies at five years post-onset. These results may reflect the high proportion of top-down therapy for CD treatment in Japan, which may indicate clinical practice aimed at early induction by advanced therapy. A meta-analysis reported that early biologic treatment led to a decreased surgery rate in patients with CD ^(14)^. The high rate of top-down therapy in this study is consistent with this finding. In the analysis by age of onset, the EO group had the highest advanced therapy-free rate among the three groups, followed by the NENP and PO groups. The EO group also showed the highest step-up rate (70.6%) among the three groups, whereas 80% of the PO group adopted the top-down strategy. Furthermore, infliximab and adalimumab were mainly selected for the PO and NENP groups, whereas vedolizumab was most commonly selected for the EO group. Older patients are typically frail and more susceptible to infection ^(15), (16), (17), (18)^. Therefore, step-up therapy with vedolizumab may be selected for the EO group in clinical practice because of its low immunosuppressive effect. In contrast, most pediatric patients receive top-down therapy and infliximab. Ongoing and chronic inflammation can lead to impairment of growth and pubertal development ^(19), (20)^. This may reflect early attempts to suppress the disadvantages of intestinal inflammation in CD. The scores of the NENP group for advanced therapy-free and top-down therapy rates were intermediate between those of the EO and PO groups. However, over 60 % of NENP patients undertook a top-down strategy. The NENP group may have been administered advanced therapies in the early phase after disease onset for the same purpose as the PO group.

Most patients treated with step-up therapy were not administered advanced therapy during their clinical course. Among patients treated with the step-up strategy, there were no differences in the cumulative advanced therapy-free rates among the three age groups. These results indicate that physicians may select an appropriate therapeutic strategy based on the patient’s age and condition. Furthermore, these results indicate that early initiation of advanced therapy may reflect high disease activity at onset, which may require advanced therapy during the clinical course.

CD activity in the PO group might be higher than that in the other two groups. The cumulative retention rate of the first advanced therapy and the systemic steroid-free rate in the PO group were the lowest among the three groups. However, systemic steroids may be deliberately avoided or minimized because of concerns regarding growth retardation and pubertal development. This clinical consideration may partly explain the distinct treatment patterns observed in this group. Furthermore, as described below, the DeSC database lacks detailed clinical data, including laboratory data and endoscopic findings, which are necessary to define disease activity. Several studies have reported that the disease behavior of PO patients with CD is similar to that of adult-onset patients ^(21), (22)^. A comparative study demonstrated that there were no differences in the prevalence of structuring and fistulizing between PO and adult onset ^(22)^. Furthermore, a review indicated that pediatric patients with CD may have higher disease activity and require more immunosuppressive therapies than adult patients ^(23)^. Although these results do not conflict with those studies, further investigations are warranted to clarify the differences in disease activity among the onset age categories. In addition, it should be noted that the observation period differed significantly across age-at-onset categories, with PO patients having the longest median follow-up. This longer follow-up may have contributed to the lower steroid-free survival and first advanced therapy retention rates observed in PO patients, as extended observation increases the likelihood of detecting treatment modifications and adverse outcomes. Therefore, differences in follow-up length should be taken into account when interpreting the results.

The analysis revealed the clinical practice of administering advanced therapies and systemic steroids in the biologic era. Systemic steroids were still used in over half of the patients. These findings indicate that the efficacy of systemic steroids remains essential and frequently required, even in the biologic era. However, a systematic review reported that concomitant steroid administration and anti-tumor necrosis factor antibodies did not improve the clinical response in the induction phase ^(24)^. Therefore, physicians should consider the effective and safe use of systemic steroids, particularly in steroid-dependent cases.

In this study, the cumulative retention rate of first advanced therapy decreased by 10-20% per year. This result was consistent with those of previous studies, which reported a loss-of-response rate of 13% for infliximab and 20.3% for adalimumab, respectively ^(25), (26)^. Furthermore, the retention rate of ustekinumab was the highest of the four tested biologics. A meta-analysis revealed that ustekinumab demonstrated similar induction efficacy in patients with CD refractory to anti-tumor necrosis factor antibodies ^(27)^. Ustekinumab may provide higher induction efficacy as first-line therapy compared with infliximab and adalimumab.

When compared with international data, several similarities and differences become apparent. A questionnaire-based survey across Asian countries showed that advanced therapies are more readily chosen as first-line induction therapy in CD, particularly in China ^(28)^. A retrospective study using electronic medical records from the United States stated that PO CD patients received advanced therapies more frequently than adult-onset patients ^(29)^. Another retrospective study using a claims database from the United States revealed that 75.8% of patients were treated with step-up therapy and 24.2% were treated with top-down therapy. Those results are similar to ours. In contrast, several studies reported that steroid dependency has declined following the introduction of biologics ^(30), (31)^, whereas our analysis revealed that more than half of patients with CD received systemic steroid. Our findings also highlight several similarities with international data, suggesting that the management of CD in Japan is likewise shifting toward biologic-centered therapy. On the other hand, our results indicate that therapeutic choices differ according to age at disease onset, implying that clinicians appropriately tailor treatment strategies based on patient age and clinical condition. While some differences compared with international reports were observed, these may be attributable to variations in healthcare systems and drug accessibility.

This study had some limitations. First, this was a retrospective study. Prospective cohort studies will be needed in the future to overcome this limitation and confirm our findings. Second, no additional patient information was available in the accessed dataset to determine disease severity, such as blood tests, endoscopic examinations, biopsy specimens, and computed tomography images. Moreover, therapeutic decisions in clinical practice are strongly influenced by disease activity and phenotype. Because these critical clinical parameters were not available in the DeSC database, we cannot rule out that the observed differences in steroid-free survival and advanced therapy retention rates, particularly in PO patients, may reflect not only age-related factors but also underlying disease activity. Therefore, the correlation between long-term outcomes of CD and disease severity remains unclear. Furthermore, there was no information on disease onset. It was assumed that patients with a new prescription of medications also had new-onset CD. As a result, patients who underwent surgery prior to initiating medical therapy were not captured. Because such patients are likely to have had more severe disease at onset, their exclusion may have biased our cohort toward those with relatively milder initial disease courses. Moreover, due to our definition of newly diagnosed CD, referral cases previously treated at other institutions may not have been excluded. This misclassification could have introduced referral bias, particularly among pediatric and younger patients who are more often referred to tertiary centers. Furthermore, the database does not capture reasons for censoring, such as insurance changes, so attrition bias that could have affected the accuracy of the long-term survival estimates cannot be fully excluded. In addition, clinical outcomes such as hospitalization and surgery were not evaluated in this study. Therefore, the cumulative retention rate of advanced therapies should not be considered a direct surrogate for disease control. Third, the study’s data may not accurately represent the population due to potential bias. The results showed a larger proportion of elderly patients than the general population. This may be because of the nature of the DeSC dataset, which contains a large amount of data on patients with CD. Although this study has a few limitations, it was possible to derive many useful findings and statistical trends from this large dataset of patients with CD. Our findings are expected to be useful in daily clinical CD practice and in future investigations.

In conclusion, the long-term outcomes and clinical practices of advanced therapy were clarified using big data. Treatment strategies for CD in Japan are influenced by the age at onset. Younger patients often receive early treatment with advanced therapies, whereas older patients are managed more conservatively. Physicians in Japan should properly evaluate each patients’ general condition and select appropriate treatment strategies and medications.

## Article Information

### Author Contributions

Conceptualization: Rintaro Moroi, Yoichi Kakuta, Hideya Iwaki, Daisuke Okamoto, Hiroshi Nagai, Yusuke Shimoyama, Takeo Naito, Hisashi Shiga. Data curation: Yoichi Kakuta. Formal analysis: Rintaro Moroi. Methodology: Rintaro Moroi, Yoichi Kakuta. Project administration: Yoichi Kakuta. Supervision: Atsushi Masamune, Visualization: Rintaro Moroi. Writing - original draft: Rintaro Moroi, Writing - review and editing, and the approval of the final manuscript: all authors

### Conflicts of Interest

None

### Ethics Approval and Patient Consent Statement

The study protocol was reviewed and approved by the Ethics Committee of the Tohoku University Graduate School of Medicine (2022-1-412). The requirement for informed consent was waived because of the anonymity of the patient data.

### Data Availability Statement

The Corresponding Author has opted not to share data because of a contract with DeSC Healthcare, Inc.

## References

[1] Matsuoka K, Kobayashi T, Ueno F, et al. Evidence-based clinical practice guidelines for inflammatory bowel disease. J Gastroenterol. 2018;53(3):305-53. 29429045 10.1007/s00535-018-1439-1 PMC5847182

[2] Colombel JF, Shin A, Gibson PR. AGA clinical practice update on functional gastrointestinal symptoms in patients with inflammatory bowel disease: expert review. Clin Gastroenterol Hepatol. 2019;17(3):380-90.e1. 30099108 10.1016/j.cgh.2018.08.001 PMC6581193

[3] Dolinger M, Torres J, Vermeire S. Crohn’s disease. Lancet. 2024;403(10432):1177-91. 38437854 10.1016/S0140-6736(23)02586-2

[4] Turner D, Ricciuto A, Lewis A, et al. STRIDE-II: an update on the Selecting Therapeutic Targets in Inflammatory Bowel Disease (STRIDE) initiative of the International Organization for the Study of IBD (IOIBD): determining therapeutic goals for treat-to-target strategies in IBD. Gastroenterology. 2021;160(5):1570-83. 33359090 10.1053/j.gastro.2020.12.031

[5] Tsui JJ, Huynh HQ. Is top-down therapy a more effective alternative to conventional step-up therapy for Crohn’s disease? Ann Gastroenterol. 2018;31(4):413-24. 29991886 10.20524/aog.2018.0253 PMC6033752

[6] Salahudeen MS. A review of current evidence allied to step-up and top-down medication therapy in inflammatory bowel disease. Drugs Today (Barc). 2019;55(6):385-405. 31250843 10.1358/dot.2019.55.6.2969816

[7] Nakase H, Uchino M, Shinzaki S, et al. Evidence-based clinical practice guidelines for inflammatory bowel disease 2020. J Gastroenterol. 2021;56(6):489-526. 33885977 10.1007/s00535-021-01784-1 PMC8137635

[8] Moroi R, Kakuta Y, Nagai H, et al. Clinical practice and outcome of patients with elderly-onset ulcerative colitis: insights from a nationwide claims database study in Japan. JGH Open. 2024;8(6):e13103. 38887512 10.1002/jgh3.13103 PMC11181292

[9] Moroi R, Kakuta Y, Nagai H, et al. Clinical utilization of generic drugs and biosimilars for ulcerative colitis treatment: insights from a nationwide database study in Japan. Inflamm Intest Dis. 2024;9(1):29-39. 38344420 10.1159/000536146 PMC10857828

[10] Moroi R, Kakuta Y, Obara T, et al. Long-term prognosis and clinical practice for new-onset ulcerative colitis in the era of biologics: a Japanese retrospective study. JGH Open. 2023;7(10):682-9. 37908295 10.1002/jgh3.12957 PMC10615172

[11] Satsangi J, Silverberg MS, Vermeire S, et al. The Montreal classification of inflammatory bowel disease: controversies, consensus, and implications. Gut. 2006;55(6):749-53. 16698746 10.1136/gut.2005.082909 PMC1856208

[12] World Health O. Men, ageing and health : achieving health across the life span. Geneva: World Health Organization; 2001. https://apps.who.int/iris/handle/10665/66941

[13] Peyrin-Biroulet L, Sandborn W, Sands BE, et al. Selecting therapeutic targets in inflammatory bowel disease (STRIDE): determining therapeutic goals for Treat-to-Target. Am J Gastroenterol. 2015;110(9):1324-38. 26303131 10.1038/ajg.2015.233

[14] Law CCY, Tkachuk B, Lieto S, et al. Early biologic treatment decreases risk of surgery in Crohn’s disease but not in ulcerative colitis: systematic review and meta-analysis. Inflamm Bowel Dis. 2024;30(7):1080-6. 37506265 10.1093/ibd/izad149 PMC11219475

[15] Sturm A, Maaser C, Mendall M, et al. European Crohn’s and colitis organisation topical review on IBD in the elderly. J Crohns Colitis. 2017;11(3):263-73. 27797918 10.1093/ecco-jcc/jjw188

[16] Kirchgesner J, Lemaitre M, Carrat F, et al. Risk of serious and opportunistic infections associated with treatment of inflammatory bowel diseases. Gastroenterology. 2018;155(2):337-46.e10. 29655835 10.1053/j.gastro.2018.04.012

[17] Bonovas S, Fiorino G, Allocca M, et al. Biologic therapies and risk of infection and malignancy in patients with inflammatory bowel disease: a systematic review and network meta-analysis. Clin Gastroenterol Hepatol. 2016;14(10):1385-97.e10. 27189910 10.1016/j.cgh.2016.04.039

[18] Cottone M, Kohn A, Daperno M, et al. Advanced age is an independent risk factor for severe infections and mortality in patients given anti-tumor necrosis factor therapy for inflammatory bowel disease. Clin Gastroenterol Hepatol. 2011;9(1):30-5. 20951835 10.1016/j.cgh.2010.09.026

[19] Motil KJ, Grand RJ, Davis-Kraft L, et al. Growth failure in children with inflammatory bowel disease: a prospective study. Gastroenterology. 1993;105(3):681-91. 8359640 10.1016/0016-5085(93)90883-e

[20] Griffiths AM, Nguyen P, Smith C, et al. Growth and clinical course of children with Crohn’s disease. Gut. 1993;34(7):939-43. 8344582 10.1136/gut.34.7.939 PMC1374230

[21] Yu YR, Rodriguez JR. Clinical presentation of Crohn’s, ulcerative colitis, and indeterminate colitis: symptoms, extraintestinal manifestations, and disease phenotypes. Semin Pediatr Surg. 2017;26(6):349-55. 29126502 10.1053/j.sempedsurg.2017.10.003

[22] Van Limbergen J, Russell RK, Drummond HE, et al. Definition of phenotypic characteristics of childhood-onset inflammatory bowel disease. Gastroenterology. 2008;135(4):1114-22. 18725221 10.1053/j.gastro.2008.06.081

[23] Mitchel EB, Rosh JR. Pediatric management of Crohn’s disease. Gastroenterol Clin North Am. 2022;51(2):401-24. 35595422 10.1016/j.gtc.2021.12.013

[24] Faleck DM, Shmidt E, Huang R, et al. Effect of concomitant therapy with steroids and tumor necrosis factor antagonists for induction of remission in patients with Crohn’s disease: a systematic review and pooled meta-analysis. Clin Gastroenterol Hepatol. 2021;19(2):238-45.e4. 32569749 10.1016/j.cgh.2020.06.036 PMC8364422

[25] Billioud V, Sandborn WJ, Peyrin-Biroulet L. Loss of response and need for adalimumab dose intensification in Crohn’s disease: a systematic review. Am J Gastroenterol. 2011;106(4):674-84. 21407178 10.1038/ajg.2011.60

[26] Harper JW, Sinanan MN, Zisman TL. Increased body mass index is associated with earlier time to loss of response to infliximab in patients with inflammatory bowel disease. Inflamm Bowel Dis. 2013;19(10):2118-24. 23863401 10.1097/MIB.0b013e31829cf401

[27] Parrot L, Dong C, Carbonnel F, et al. Systematic review with meta-analysis: the effectiveness of either ustekinumab or vedolizumab in patients with Crohn’s disease refractory to anti-tumour necrosis factor. Aliment Pharmacol Ther. 2022;55(4):380-8. 34854100 10.1111/apt.16714

[28] Song EM, Na SY, Hong SN, et al. Treatment of inflammatory bowel disease-Asian perspectives: the results of a multinational web-based survey in the 8th Asian Organization for Crohn’s and Colitis meeting. Intest Res. 2023;21(3):339-52. 37533265 10.5217/ir.2022.00135 PMC10397553

[29] Kurowski JA, Milinovich A, Ji X, et al. Differences in biologic utilization and surgery rates in pediatric and adult Crohn’s disease: results from a large electronic medical record-derived cohort. Inflamm Bowel Dis. 2021;27(7):1035-44. 32914165 10.1093/ibd/izaa239

[30] Chen NY, Chuang CH, Chang YC, et al. Epidemiology and steroid dependency reduction in Crohn’s disease during the biologics era: a nationwide population-based study. Adv Ther. 2025;42(9):4318-34. 40549269 10.1007/s12325-025-03245-0 PMC12394366

[31] Holko P, Kawalec P, Pilc A. Impact of biologic treatment of Crohn’s disease on the rate of surgeries and other healthcare resources: an analysis of a nationwide database from Poland. Front Pharmacol. 2018;9:621. 29942260 10.3389/fphar.2018.00621 PMC6004509

